# Layer by Layer Mesoporous Silica-Hyaluronic Acid-Cyclodextrin Bifunctional “Lamination”: Study of the Application of Fluorescent Probe and Host–Guest Interactions in the Drug Delivery Field

**DOI:** 10.3390/ma11091745

**Published:** 2018-09-17

**Authors:** Kun Nie, Qi An, Jeffrey I. Zink, Xiang Yu, Yihe Zhang

**Affiliations:** 1 Beijing Key Laboratory of Materials Utilization of Nonmetallic Minerals and Solid Wastes, National Laboratory of Mineral Materials, School of Materials Science and Technology, China University of Geosciences (Beijing), Beijing 100083, China; nk@cugb.edu.cn (K.N.); an@cugb.edu.cn (Q.A.); 2 Department of Chemistry and Biochemistry and California Nano Systems Institute, University of California, Los Angeles, CA 90095, USA; zink@chem.ucla.edu

**Keywords:** peptide, lamination, mesoporous silica, drug delivery, host–guest interactions

## Abstract

The layer-by-layer technique was exploited to adjust the magnitude of the host–guest interactions between adamantane and cyclodextrin. The effect depends on numerous complex and changeable growth profiles of the films and the number of bilayers. These composite films of mesoporous silica nanoparticles and hyaluronic acid–cyclodextrin(HA-CD) were constructed to load the fluorescent dyes and peptides. The release rates of these molecules would decrease with an increase in the number of layers. A laser scanning confocal microscope was utilized to obtain the diffusion coefficient of fluorescein isothiocyanate. Hybrid films could be applied to increase the loading of different kinds of molecules and could also be integrated into the lamination to delay the rate of release.

## 1. Introduction

The layer-by-layer (LbL) technique is a facile method to prepare hybrid films with a structural control, at the nanoscopic scale [1,2,3,4]. It is among the most widely used techniques to construct the organic–inorganic hybrid films [5,6,7]. Due to the rich functionalities of an integration of the organic and inorganic parts [8,9,10], hybrid films that simultaneously incorporate polymeric species and inorganic nanoparticles are favored all over the world [11,12]. In general, hybrid films which incorporate inorganic nanoparticles are prepared either by using a covalent cross-linkage or by the non-covalent interactions between these polymers and inorganic nanoparticles [13,14,15]. Non-covalently assembled hybrid films could be achieved by using various supramolecular interactions [16,17,18,19,20], including electrostatic interactions [21,22,23], hydrophobic interactions [24,25], host–guest interactions [26,27,28,29], a hydrogen bonding, and a coordination bonding [30,31]. Among them, host–guest interactions play an important role in the field of diffusion of fluorescent molecules, which is widely used in the field of drug delivery. Therefore, improvements in the diffusion of fluorescent molecules are valuable and could provide an insight into the host–guest interactions in diverse, advanced functional materials [32,33,34]. For the assessment of host–guest diffusion, fluorescence recovery after photobleaching (FRAP) is the most popular methodology [33,35,36,37].

According to the literature, biocompatible films are a promising class of materials in the field of drug diffusion. Due to the high biocompatibility, hyaluronic acid is usually selected as one of the ingredients of the film. Furthermore, hyaluronic acid has many other properties that meet the requirements of the experiment [38]. Repeating-disaccharides of *N*-acetyl-D-glucosamine and β-glucuronic acid are the main components of hyaluronic acid (HA) [39,40]. Taking advantage of the cell-surface receptors, HA can anchor to the cell surface effectively. HA hydrogel is widely distributed in glycosaminoglycan superstructure complexes, and in the synovial fluid [41]. As shown in the literature, HA could contribute as an outstanding delivery device to the cartilage repair and regeneration processes [42]. Due to its potential applications, HA has been used in many innovative fields such as stimuli-responsive gels, biosensing devices, gene carriers, and so on. Moreover, there are a lot of studies regarding the interactions between HA and supramolecular structures [43]. Cyclic oligosaccharide, with a hydrophilic outer surface and a lipophilic central cavity, which could be abbreviated to CD, is frequently used among supramolecular structures [44]. However, the CD molecules are large and are impermeable to lipophilic membranes, owing to its multitudinous hydrogen donors and acceptors. Moreover, CD has also been quite extensively used as a complexing agent, in pharmaceutical fields, to improve the aqueous solubility, bioavailability, and stability of the insoluble drugs [43,44].

In this study, a facile strategy was reported to construct laminated multilayer films material, by using an hyaluronic acid-cyclodextrin(HA-CD) combination, which could effectively preserve various types of peptides. Herein, “lamination” was the technique of manufacturing the same kinds of continuous membrane structures, in multiple layers, so that the composite films can achieve various improved functions, a high stability, and other properties, from the use of the different multilayers. Mesoporous silica nanoparticles (MSN) act as the peptide–nanocontainer and are embedded within the functional lamination multilayers, which are built via electrostatic interactions. Due to the delicate structural construction, the laminated multilayer films can modulate the magnitude of supramolecular interactions. By controlling the number of laminated multilayers, the spatial-temporal release profiles of the peptides could be adjusted. To further demonstrate the functions of the reported peptide immobilization strategy, a supramolecular force-delay molecular release was chosen as an example. It was found that that the reported method of “lamination” fabrication could control the release of peptides via the preparation of supramolecular functional films.

## 2. Materials and Methods

### 2.1. Materials and Instruments

The following chemicals were used as received: mono-(6-amino-6-deoxy)-beta-cyclodextrin was bought from Shandong Binzhou Zhiyuan Biotechnology Co., Ltd (Binzhou, China). Hyaluronic acid (HA, 95%), fluoresceinisothiocyanate (FITC, purity > 90%, Mw = 389.40), fluorescein sodium, phosphate buffer solution (PBS) and Arginylglycylaspartic acid(RGD) peptides-FITC (FITC-RGD & FITC-RGD-Ad) modified with fluoresceinisothiocyanate were obtained from Sinopharm Chemical Reagent Beijing Co., Ltd (Beijing, China). Cetyltrimethylammonium bromide surfactant (CH_3_(CH_2_)_15_N(CH_3_)_3_Br, CTAB), tetraethoxysilane (TEOS) and Poly(allylamine hydrochloride) (PAH, Mw = 15,000) were purchased from Sigma-Aldrich. 4,4′-diazostilbene-2,2′-disulfonic acid disodium salt (DAS), *N*-Hydroxysuccinimide (NHS) and 1-(3-Dimethylaminopropyl)-3-ethylcarbodiimide hydrochloride (EDC) were purchased from Tokyo Chemical Industry (Shanghai) Development Co., Ltd (Shanghai, China). Only Ultra-pure water was used, in this study.

### 2.2. General UV-vis, Fluorescence Spectroscopy, Transmission Electron Microscopy (TEM) and Brunauer-Emmett-Teller (BET) Surface Area Analysis

UV-vis spectra were obtained on the Hitachi U-3900 spectrophotometer (Hitachi, Tokyo, Japan). Surface morphologies of polyelectrolyte multilayers were characterized with Transmission electron microscopy (TEM, Tecnai T12, Field Electron and Ion Company, FEI, Hillsboro, OR, USA). TEM experiments were carried out on a Titan S/TEM (Field Electron and Ion Company, FEI) microscope (Hillsboro, OR, USA). Fluorescence recovery after photobleaching (FRAP, A1Rsi, Nikon, Tokyo, Japan) images were obtained by laser scanning confocal microscope (A1Rsi, Nikon, Tokyo, Japan). Proton Nuclear Magnetic Resonance (^1^H NMR) spectrum was obtained on Bruker Avance III 400 MHz WB (Bruker, Baden, Switzerland). The Brunauer-Emmett-Teller(BET)-Barret-Joyner-Halenda(BJH) BET-BJH data were obtained from the Autosorb-iQ2 (Quantachrome, Boynton Beach, FL, USA).

### 2.3. The Synthesis of HA-CD Hydrogels

At first, in a 100 mL erlenmeyer flask, 0.3 g of sodium hyaluronate and 50 mL of morpholinoethanesulfonic buffer (MES) were added and vigorously stirred for 6 h. Subsequently, 0.285 g of NHS and 0.342 g of EDC were added into the erlenmeyer flask. Then the mixture was stirred for another hour. To this mixture, 0.1686 g of amino cyclodextrin was added, followed by stirring for 24 h. The experimental recipe and the sample code are listed in Table S1. As shown in Figure S1, the peak at 5.10 ppm represents the proton of amido(NH) (5.10 ppm) group. According to the ^1^H NMR (400 MHz, D_2_O) data, the grafting rate of HA-CD was 4.71%.

### 2.4. The Preparation of Mesoporous Silica Nanoparticles (MSN)

Mesoporous silica nanoparticles were synthetized as reported [45]. 2.0 g of CTAB (sixteen alkyl three methyl ammonium bromide), 7.0 mL NaOH (aq, 2.0 mol L^−1^), and 480 g H_2_O was mixed in the erlenmeyer flask, for 30 min at 80 °C, with stirring. Subsequently, 8.31 g TEOS was added into the mixture. After two min, white solid appeared. The product was heated for another 2 h at 80 °C, centrifuged, washed with plenty of ultra-pure water, and dried in a vacuum oven for 12 h. Afterward, another reaction was carried out at 60 °C for the extraction of CTAB, by adding the synthesized product to the mixture of 120 mL ethanol and 1.0 mL concentrated hydrochloric acid, for 8 h. After that, the sample was centrifuged three times, washed with ultra-pure water and ethanol three times, and then dried in a vacuum oven. The mesoporous silica nanoparticles were about 100 nm, as observed from the TEM images seen in Figure S2. The surface area of the MSNs was 646.715 m^2^ g^−1^ and the pore diameter was 3.4 nm, which were characterized by the BET-BJH instrument.

### 2.5. The Modification of Quartz Sheets

The modification of quartz sheets (1 × 3 cm^2^), to get abundant hydroxyl groups, was implemented via the following strategy: Sixteen quartz sheets were immersed into an acid solution (concentrated H_2_SO_4_/H_2_O_2_ (v:v = 7:3)) for 6 h, followed by a washing with a lot of ultra-pure water.

### 2.6. The Preparation of (PAH/SiO_2_)_n_/(PAH/HA–CD)_n_ Layer-by-Layer Films

The preparation of (PAH/SiO_2_)*_n_*/(PAH/HA–CD)*_n_* multilayers was indicated by UV-vis spectra. (They were similar to that shown in Figure S3). The number of layers was controlled by the assembly time of each layer, as well as the total assembly time for all the layers together. The LbL process of (PAH/SiO_2_)*_n_* films was implemented as follows: Firstly, quartz substrates rich in hydroxyl group were soaked into PAH solution (aq, Mw = 15,000, 1 mg mL^−1^, pH = 9.0), for 30 min, washed with ultra-pure water three times, and then dried with nitrogen (N_2_); because the surface of the PAH, the SiO_2_ and the HA-CD were all negatively charged. Then, the quartz substrates were immersed in the dispersion of SiO_2_ (1 mg mL^−1^), for 30 min, washed three times with ultra-pure water, then fully dried under N_2_. At last, the above several steps were repeated, in turn, until the expected number of multilayer films was achieved. The LbL process of (PAH/HA-CD)*_n_* was similar, except that, an HA-CD (0.1 mg mL^−1^) solution was used in place of the SiO_2_, in the experiment.

### 2.7. Adsorption and Sustained Release of Peptide

For the sustained-release experiment, substrates covered with multilayer films were immersed in a peptide-FITC solution (0.1 mg mL^−1^), for 24 h. Subsequently, these quartz substrates were dried under nitrogen. Then, these quartz substrates were soaked in a phosphate buffer solution (PBS) for sustained release. The absorbance peak of FITC was around 480 nm. The amount of released peptide-FITC was monitored by a UV-vis spectroscopy.

### 2.8. The Calculation Method of Diffusion Coefficient

As a result of the above considerations, it was feasible to calculate the diffusion coefficient (*D*) from the recovery half-time, as stated above, in the groundbreaking paper for a Gaussian dot and a uniform circular dot [31,33,46,47]. The size of the dot was marked as *ω* and *D* could be calculated based on the following Equation:

(1) $$D=\gamma \left(\frac{\omega^{2}}{4t_{1/2}}\right)$$

where the *ω* was 80 µm for the experiment, *t*_1/2_ was half-life, *γ* was a correction factor which depends on the area of the region of bleaching, and the amount of the photobleaching and would be identified as 0.88 for a uniform circular spot.

## 3. Results and Discussion

**Scheme 1:**
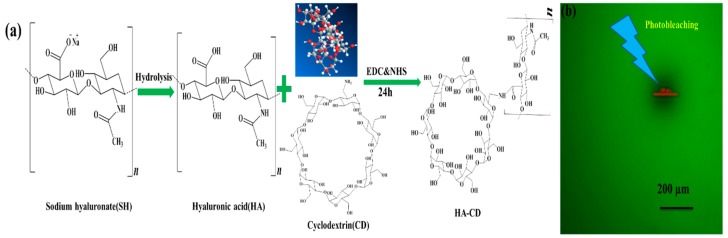
(**a**) The synthesis process of HA-CD hydrogel; (**b**) the prepared HA-CD hydrogel was used in the fluorescence recovery after the photobleaching (FRAP) experiment.

**Scheme 2:**
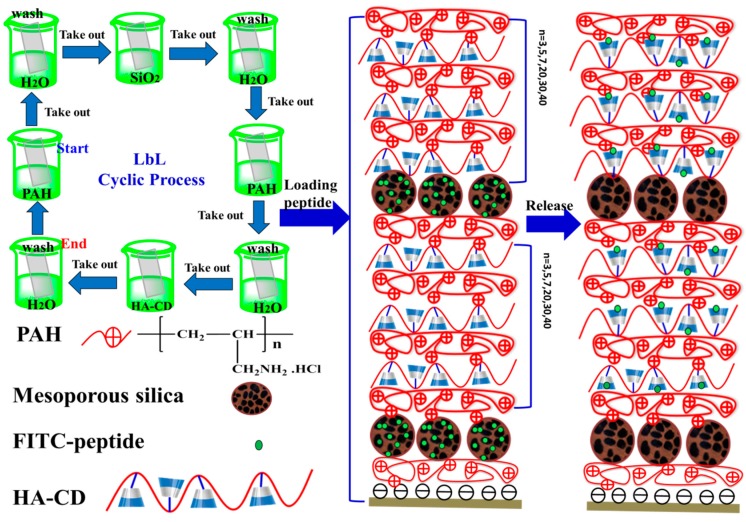
Illustration of the construction of the short peptide retention and the release of multilayers, using the Layer-by-layer (LbL) technique: Multilayer films were prepared on the surface of the quartz plate via a layer-by-layer self-assembly technique. The prepared multilayer films were used to load the peptides. Both the laminated layer and the mesoporous silica reservoir were indispensable in obtaining the functional laminations. The capping layers were (HA-CD/Poly(allylamine hydrochloride (PAH))*_n_* woven multilayers.

As illustrated in Scheme 1, aminocyclodextrins were grafted onto the hyaluronic acid through a series of chemical reactions. ^1^H NMR spectroscopy was used to determine the degree of grafting of HA-CD. The graft ratio was calculated by using the integral area of the ^1^H NMR spectrum. As shown in Figure S1, in ^1^H NMR the peak at 5.10 ppm represents the proton of NH (5.10 ppm) group and its integral area was 0.11. Therefore, the integral area of each hydrogen atom of the aminocyclodextrin was 0.11/7. Similarly, the peak at 2.05 ppm represents the proton of CH_3_ (2.05 ppm) group and its integral area was 1.00. Therefore, the integral area of each hydrogen atom of hyaluronic acid was 1/3. Grafting ratio was obtained by dividing the integral area of each hydrogen atom of amino cyclodextrin by the integral area of each hydrogen atom of hyaluronic acid. So, the grafting rate HA-CD was 4.71%. The sodium hyaluronate solution (300 mg in 50 mL MES buffer) was mixed with mono-(6-amino-6-deoxy)-beta-cyclodextrin, EDC, and NHS, followed by stirring for 24 h (Scheme 1a). The mixture was filtered with a dialysis bag to remove the impurities and freeze-dried to yield a white flocculent solid (HA-CD), which was analyzed using ^1^H NMR spectroscopy. The prepared HA-CD was dissolved and then applied as a medium in the fluorescence recovery after the photobleaching experiment. Fluorescence recovery after photobleaching was based on a facile method. Photobleaching meant that the small fluorescent molecules in a fluorescence-labeled sample were irreversibly bleached by a high-intensity pulse of light with a limited region; the process is illustrated in Scheme 1b. The area of the region of interest (ROI) was 20084.46 µm^2^ and the diameter (*D*) of this region was about 160 μm, in the experiment. The photobleaching time was only 1 s, in the experiment. The potent peptide retention laminated multilayer films reported here were constructed via integrating MSN, as the peptide nanocontainer, with the laminated layers, to adjust the magnitude of supramolecular interactions between the adamantane (Ad) and the CD. The realization of the functionalities was inseparable from the molecules of the nanocontainer and the laminated layer (Scheme 2).

**Figure 1:**
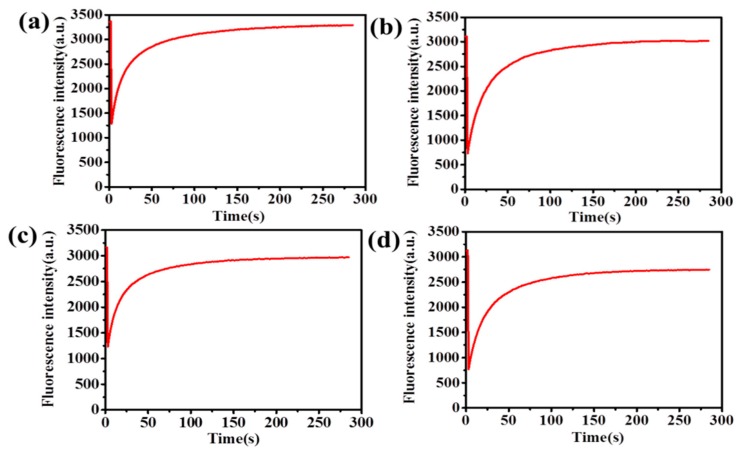
Schematic representations of the fluorescence recovery after photobleaching (FRAP) experiments on sodium fluorescein (**a**) the FRAP image of pure hyaluronic acid (HA) (3 wt.%); (**b**) the FRAP image of HA-CD (3 wt.%, grafting rate 4.71%); (**c**) the FRAP image of pure HA (4 wt.%); (**d**) the FRAP image of HA-CD (4 wt.%, grafting rate 4.71%).

**Figure 2:**
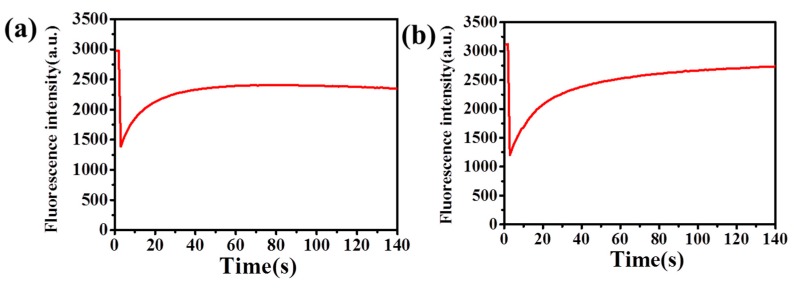
The schematic representations of the FRAP experiments on fluoresceinisothiocyanate (FITC) (**a**) the FRAP image of pure HA (3 wt.%), (**b**) the FRAP image of HA-CD (3 wt.%, grafting rate 4.71%).

The results of the FRAP experiments showed that fluorescence intensity in the target area decreased suddenly and could be seen in the recovery curve, in Figure 1 and Figure 2. The unbleached fluorophores would spread to the bleaching area while the bleached fluorophores would diffuse out. It could be concluded that the fluorescence intensity would recover, depending on the mobility of the molecules, after the final photobleaching. The exact quantitative information on the molecular dynamics, such as the diffusion, could be summarized by analyzing the time change of the fluorescence recovery with an appropriate mathematical model. Interestingly, the fluorescence intensity was dynamically changeable and could return to the original, after a sufficiently long time. Thus, the bleached molecule was immobile.

As shown in Figure 1 and Table S2, fitting Equation 1 to the FRAP experiment data permitted an estimation of the diffusion coefficient (*D*); the *D*(a) was 34.4338 µm^2^ s^−1^ (Figure 1a); the *D*(b) was 32.4881 µm^2^ s^−1^ (Figure 1b); the *D*(c) was 34.1532 µm^2^ s^−1^ (Figure 1c); the *D*(d) was 28.9492 µm^2^ s^−1^ (Figure 1d). According to the diffusion coefficient, the release rate of sodium fluorescein was fastest in the pure HA (3 wt.%), and slowest in the HA–CD hydrogels (3 wt.%, grafting rate 4.71%). When the wt.% of hydrogels were the same, the release rates of fluorescent molecules were different. CDs can form complexes with hydrophobic cavity, benefitting from the features of the hydrophobic environment inside, and the hydrophilic environment outside. Thus, it was possible to form the water-in-oil (W/O) structure. Therefore, the release of the fluorescent probe might have been delayed or even blocked. It indicated that the release speed of sodium fluorescein was easily affected, under the same experimental conditions. Despite the fact that the FRAP phenomenon was obvious, the sodium fluorescein was not the best FRAP agent in this experiment. But diffusion coefficient showed that Equation 1 was quite applicable to get appropriate results for the experimental diffusion coefficient of fluorescent probe. Useful information, such as the mobility of the FITC labeled molecule or other structure of nanomaterials in which the small molecule diffuse could be achieved by the FRAP experiments. In other words, fluorescence molecular diffusion data can be obtained in the FRAP experiments. For fluorescence recovery after photobleaching experiments, the fluorescent probe might have had intermediate photo stability. On one hand, it should not have been too difficult to bleach; on the other hand, it should have had sufficient light stability to allow imaging of the fluorescence recovery process.

The most commonly applied hydrophilic fluorophore was fluoresceinisothiocyanate (FITC). It could easily be attached to proteins and polysaccharides and had a good balance between photostability and instability. To verify the generality of our reported FITC, the fluorescent molecules, FITC, was used in the FRAP experiment (Figure 2). FRAP was entirely based on fluorescence. Therefore, it is necessary to briefly outline the fluorescence-related phenomenon, that demand attention, when performing or analyzing FRAP experiments.

Figure 2a and Table S3 shows that the diffusion coefficient *D*(a) was 30.2861 µm^2^ s^−1^, and Figure 2b shows that the *D*(b) was 30.2847 µm^2^ s^−1^. It shows that both the data were very close, under different conditions. Which fluorescent probe to choose for the FRAP experiments depended on the available excitation source, the hydrophilic or hydrophobic properties of the medium in which the fluorescent probe had to be soluble, and the chemical ways that were available to attach the fluorophore to the molecule of interest. Fluorescein was acquired by the reactive form of FITC, due to the isothiocyanate group, and it was used as a label-specific molecule by attaching to larger molecules, such as dextran or other large complexes. In the method discussed above, FITC and FITC-derivative would have been a good fluorescent probe, in the experiment of drug delivery.

**Figure 3:**
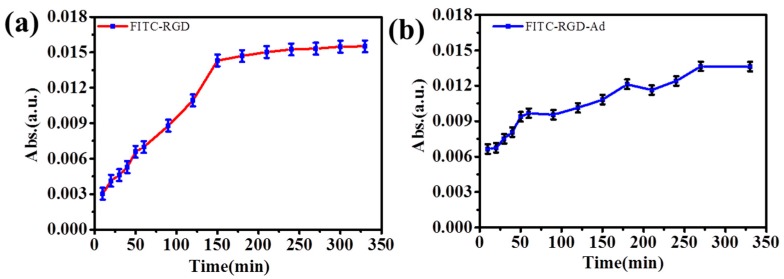
The release profiles of (**a**) FITC-RGD and (**b**) FITC-RGD-Ad from (PAH/SiO_2_)_1_/(PAH/HA-CD)_3_/(PAH/SiO_2_)_1_/(PAH/HA-CD)_3_ multilayer films.

From the above considerations, the FITC-labeled peptide was used as a model drug. Figure 3 and Table S4 show the peptide release profiles from hydrogels, loaded with varying kinds of bio-peptide. Figure 3a clearly shows that the drug release rate of FITC-RGD was faster than the rate of FITC-RGD-Ad. The release curve clearly showed that the release time of FITC-RGD was about 150 min (Figure 3a); however, the release time of FITC-RGD is about 250 min (Figure 3b). The release profile in Figure 3a was smooth, in clear comparison, the plot of FITC-RGD-Ad was fluctuant, that is, first it increased quickly, then it increased slowly, in the (PAH/SiO_2_)_1_/(PAH/HA-CD)_3_/(PAH/SiO_2_)_1_/(PAH/HA-CD)_3_ multilayer films. Structurally, in a multilayer film, each layer of mesoporous silica layers was assembled and then multiple layers of the same HA-CD polymer film were assembled. There were host–guest interactions between cyclodextrin and adamantane. Therefore, when the adamantane-labeled peptide passed through the HA-CD multilayer films, its release rate became slower. When a normal peptide passed through HA-CD multilayer films, its release rate did not slow down.

**Figure 4:**
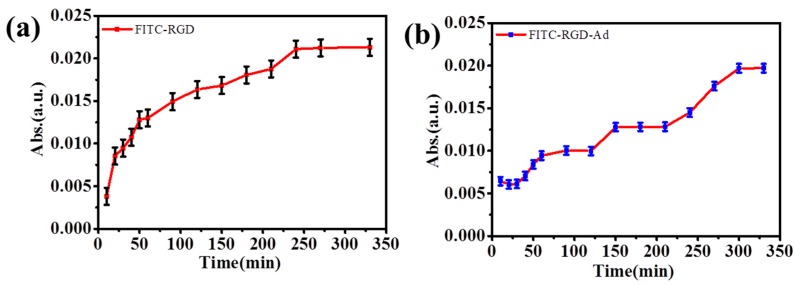
The release curves of (**a**) FITC–RGD and (**b**) FITC-RGD-Ad from (PAH/SiO_2_)_1_/(PAH/HA-CD)_5_/(PAH/SiO_2_)_1_/(PAH/HA-CD)_5_ multilayer films.

**Figure 5:**
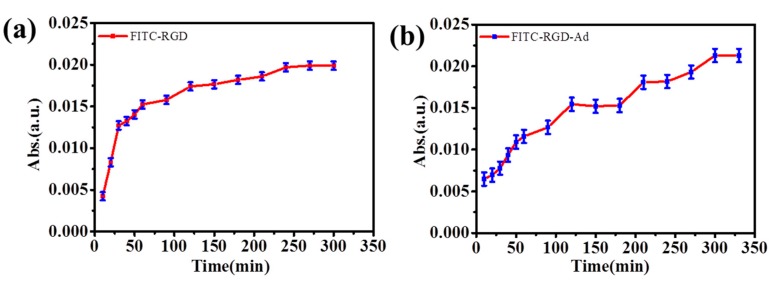
The release curves of (**a**) FITC-RGD and (**b**) FITC-RGD-Ad from (PAH/SiO_2_)_1_/(PAH/HA-CD)_7_/(PAH-SiO_2_)_1_/(PAH/HA-CD)_7_ multilayer films.

Thus, the hydroxyl groups were on the outer edges, only while the internal cavity contained only hydrogen atoms, and the oxygen bridged the result from the host-guest interactions between the cyclodextrin and the adamantane, emerging as three-dimensional structures. The CDs possessed the external hydrophilic surface and the hydrophobic central cavity, as a result of the interactions above. A similar phenomenon also appeared in (PAH/SiO_2_)_1_/(PAH/HA-CD)_5_/(PAH/SiO_2_)_1_/(PAH/HA-CD)_5_ (Figure 4 and Table S4) multilayer films and (PAH/SiO_2_)_1_/(PAH/HA-CD)_7_/(PAH/SiO_2_)_1_/(PAH/HA-CD)_7_ (Figure 5) multilayer films. The reason for the delayed effect of the laminated films was that the host–guest supramolecular interactions in the release process increased with an increase in the number of laminations. The multilayers of (PAH/HA-CD)_5_ lamination were only able to release FITC-RGD for 200 min, however, FITC-RGD-Ad required nearly 290 min. Compared with (PAH/SiO_2_)_1_/(PAH/HA-CD)_3_/(PAH/SiO_2_)_1_/(PAH/HA-CD)_3_ multilayer films, the released amounts of FITC-RGD and FITC-RGD-Ad were much higher because the number of laminations was bigger than before. The host-guest interactions would have naturally increased, due to this.

As a step further, (PAH/SiO_2_)_1_/(PAH/HA-CD)_7_/(PAH-SiO_2_)_1_/(PAH/HA-CD)_7_ multilayer films were prepared to confirm this phenomenon. As illustrated in Figure 5 and Table S4, the release time of FITC-RGD-Ad was the longest, up to nearly 330 min, which was longer than any other multilayer films, however, FITC-RGD-Ad requires only 270 min. The curve changes further demonstrated that the supramolecular force did increase with the increase in the number of laminations. As the number of HA-CD lamination layers increased, the release rate of the fluorescent probe increased and then decreased more significantly, during the release process.

**Figure 6:**
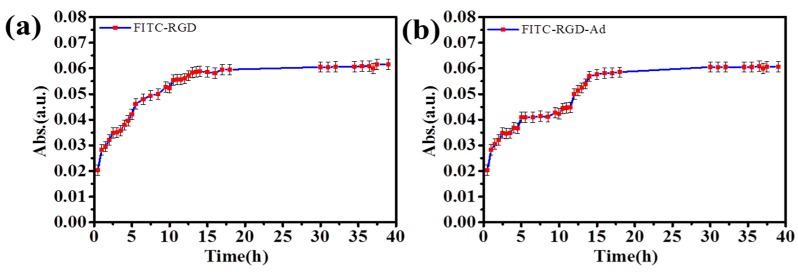
The release plots of (**a**) FITC-RGD and (**b**) FITC-RGD-Ad from (PAH/SiO_2_)_1_/(PAH/HA-CD)_20_/(PAH-SiO_2_)_1_/(PAH/HA-CD)_20_ laminated multilayer films.

**Figure 7:**
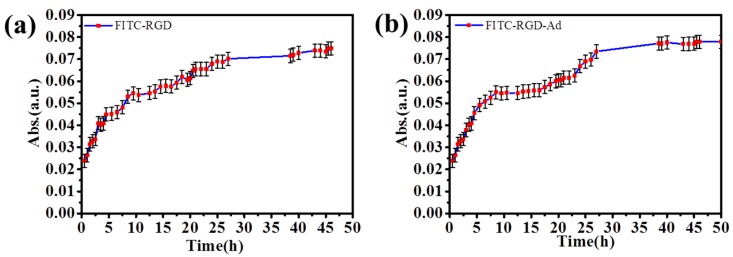
The release plots of (**a**) FITC–RGD and (**b**) FITC–RGD–Ad from (PAH/SiO_2_)_1_/(PAH/HA–CD)_30_/(PAH/SiO_2_)_1_/(PAH/HA–CD)_30_ laminated multilayer films.

**Figure 8:**
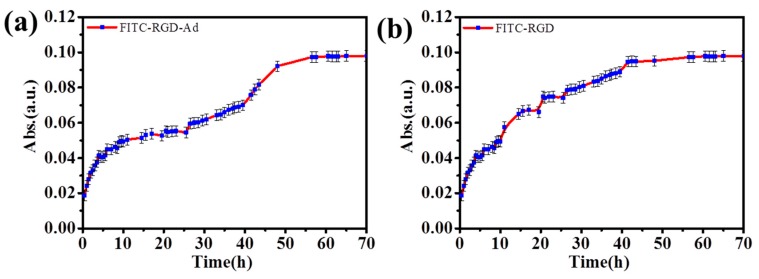
The release curves of (**a**) FITC–RGD and (**b**) FITC–RGD–Ad from (PAH/SiO_2_)_1_/(PAH/HA–CD)_40_/(PAH/SiO_2_)_1_/(PAH/HA–CD)_40_ laminated multilayer films.

Subsequently, in the release process of fluorescent probe it was wondered whether these (PAH/SiO_2_)_1_/(PAH/HA-CD)_20_/(PAH-SiO_2_)_1_/(PAH/HA-CD)_20_,(PAH/SiO_2_)_1_/(PAH/HA-CD)_30_/(PAH-SiO_2_)_1_/(PAH/HA-CD)_40_ and (PAH/SiO_2_)_1_/(PAH/HA-CD)_40_/(PAH-SiO_2_)_1_/(PAH/HA-CD)_40_ lamination films presented a higher host–guest interactions, compared with the (PAH/SiO_2_)_1_/(PAH/HA-CD)_7_/(PAH-SiO_2_)_1_/(PAH/HA-CD)_7_ lamination films, wherein the phenomena existed, and was conspicuous in the experiment, according to Figure 6, Figure 7 and Figure 8. When the number of (PAH/HA-CD)*_n_* laminated multilayer films was the same, the release time of FITC-RGD-Ad was longer than FITC–RGD. So, it was important to study the influence of the laminated films on the delayed release of FITC-RGD-Ad and the FITC-RGD-Ad from the multilayers. As shown in Figure 6a and Table S5, the release time of FITC-RGD was about 15 h and the release time of the FITC-RGD-Ad was about 27 h (Figure 6b). In terms of Figure 6b and Table S5, the release rate of FITC-RGD-Ad had a sharp increase within the first 15 h and then showed slow increases. It indicated that host–guest interactions were more prominent. The significant effect also appeared in Figure 7b and Figure 8a. As was described in Figure 7 and Table S5, the release time of FITC-RGD was about 27 h (Figure 7a) and the release time of the FITC–RGD–Ad was about 40 h (Figure 7b). The release speed of FITC-RGD-Ad and FITC-RGD slowed down, and the number of (PAH/HA-CD)*_n_* superstratum also increased. Moreover, the release speed of FITC-RGD-Ad showed a sharp increase, first, and then a slow increase, and the release time was longer than the release time of FITC-RGD. It took about 55 h for the (PAH/SiO_2_)_1_/(PAH/HA-CD)_40_/(PAH/SiO_2_)_1_/(PAH/HA-CD)_40_ laminated multilayer films to achieve the saturated release of FITC-RGD-Ad, and about 40 h for FITC–RGD (Figure 8 and Table S5). The release profiles of FITC-RGD-Ad from (PAH/SiO_2_)_1_/(PAH/HA-CD)*_n_*/(PAH/SiO_2_)_1_/(PAH/HA-CD)*_n_* (*n* = 20, 30 and 40) laminated multilayers obviously deviated from the regular release curves of loaded layer-by-layer multilayers films. The heterogeneous nature of our composite laminated multilayer films might have led to these deviations. These results demonstrated that the release profiles of FITC-RGD-Ad were effectively adjusted by the laminated multilayers, in this experiment.

**Figure 9:**
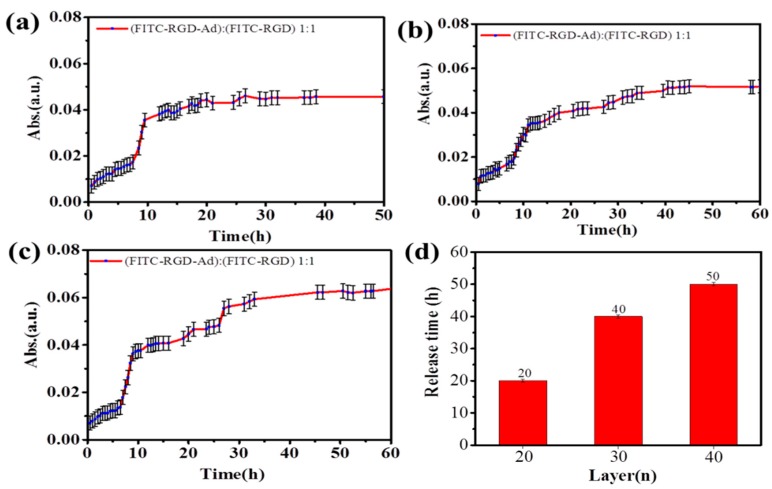
The release profiles of mixed solution of FITC–RGD and FITC–RGD–AD (V:V = 1:1) from (**a**) (PAH/SiO_2_)_1_/(PAH/HA–CD)_20_/(PAH-SiO_2_)_1_/(PAH/HA–CD)_20_; (**b**) (PAH/SiO_2_)_1_/(PAH/HA–CD)_30_/(PAH/SiO_2_)_1_/(PAH/HA–CD)_30_; (**c**) (PAH/SiO_2_)_1_/(PAH/HA–CD)_40_/(PAH/SiO_2_)_1_/(PAH/HA–CD)_40_ laminated multilayer films; (**d**) the release time of different laminated multilayer films (n = 20, 30 and 40).

To demonstrate the generalization of our reported laminated multilayer films strategy, the different kinds of laminated multilayers were immersed into a mixed solution of FITC–RGD and FITC–RGD–Ad (V:V = 1:1), respectively, as depicted in Figure 9. Encouraged by the desirable optical response in only FITC–RGD or FITC–RGD–Ad aqueous solution, the capability of the mixed fluorescent probe was then explored to monitor the FITC in a phosphate buffer saline (PBS), using a Hitachi U-3900 spectrophotometer. As illustrated in Figure 9, the release time of the fluorescent probe was around 20 h, for the (PAH/SiO_2_)_1_/(PAH/HA–CD)_20_/(PAH-SiO_2_)_1_/(PAH/HA–CD)_20_ laminated multilayer, to obtain a saturated release of fluorescent probe (Figure 9a), around 40 h for the (PAH/SiO_2_)_1_/(PAH/HA–CD)_30_/(PAH/SiO_2_)_1_/(PAH/HA–CD)_30_ (Figure 9b), and over 50 h for the (PAH/SiO_2_)_1_/(PAH/HA–CD)_40_/(PAH/SiO_2_)_1_/(PAH/HA–CD)_40_ laminated multilayer films (Figure 9c), in the release process.

As shown in Figure 9, at first, the release rate of the peptide increased slowly and then increased rapidly. The quick release time of fluorescent probe was not long. However, as the number of (PAH/HA–CD)_n_ laminated multilayer films increased, the release speed of mixed fluorescent probes slowed down, and the release time also increased. Because there was no host–guest interactions between the FITC–RGD and HA–CD, the release speed of the FITC–RGD was not delayed by the supramolecular force since the solution was a mixed solution of the FITC–RGD and FITC–RGD–Ad. First, due to host–guest interactions, the release rate of the fluorescent probe in the solution was slower. When passing through the mesoporous silica region, both fluorescent molecules were not affected by the host and guest interactions, resulting in a rapid increase in the release rate. Subsequently, when the fluorescent probe passed through the HA film, the release rate of the fluorescent probe decreased again. The reason was that there was a kind of a host–guest interaction between the FITC–RGD–Ad and the HA–CD. It indicated that the mixed fluorescent probe was an effective method for laminated multilayer films. The more the laminated multilayer films, the greater the host–guest interactions. The reason behind the masking of the fluorescent probe was the host–guest interactions between the HA–CD and the Ad, delaying the release speed of fluorescent molecules, therein.

## 4. Conclusions

In the present work, a facile strategy to construct laminated multilayer films embedded with mesoporous silica nanoparticles has been developed. Mesoporous silica nanoparticles acted as the general fluorescent probe and the peptide containers. The layer-by-layer technique was adopted for preparing the laminated multilayer films since it could modulate the magnitude of supramolecular interactions. Compared with conventionally prepared LbL multilayer films, the laminated composite films obtained in this study remarkably demonstrated enhanced supramolecular interactions, during the release of peptides in numerous lamination environments, and they could be reused with homologous performances. The functionalities of peptides incorporated into these nanocontainers, which were made of laminated multilayer films, could be achieved by their gradual diffusion into the contacting solution. The diffusion speed of the contained peptides changed, depending on the number of the lamination layers. Functional laminations were further prepared to further discuss whether the laminated multilayers could be incorporated and could act as a functional agent, in the building of smart devices. It is desired that the report can push forward the utilization of peptides-based functional laminations, in the future.

## Supplementary Materials

The following are available online at http://www.mdpi.com/1996-1944/11/9/1745/s1.
