# Supplementary material for: Layer by Layer Mesoporous Silica-Hyaluronic Acid-Cyclodextrin Bifunctional “Lamination”: Study of the Application of Fluorescent Probe and Host–Guest Interactions in the Drug Delivery Field
Source: Materials (Basel). 2018 Sep 17;11(9):1745. doi: 10.3390/ma11091745 (PMC6164273; doi:10.3390/ma11091745)
Supplement: Supplementary file 1 [file materials-11-01745-s001.pdf]

Supporting Information

Layer by Layer Mesoporous Silica-Hyaluronic Acid-Cyclodextrin Bifunctional "Lamination": Study the Application of Fluorescent Probe and Host-Guest Interaction in Drug Delivery Field

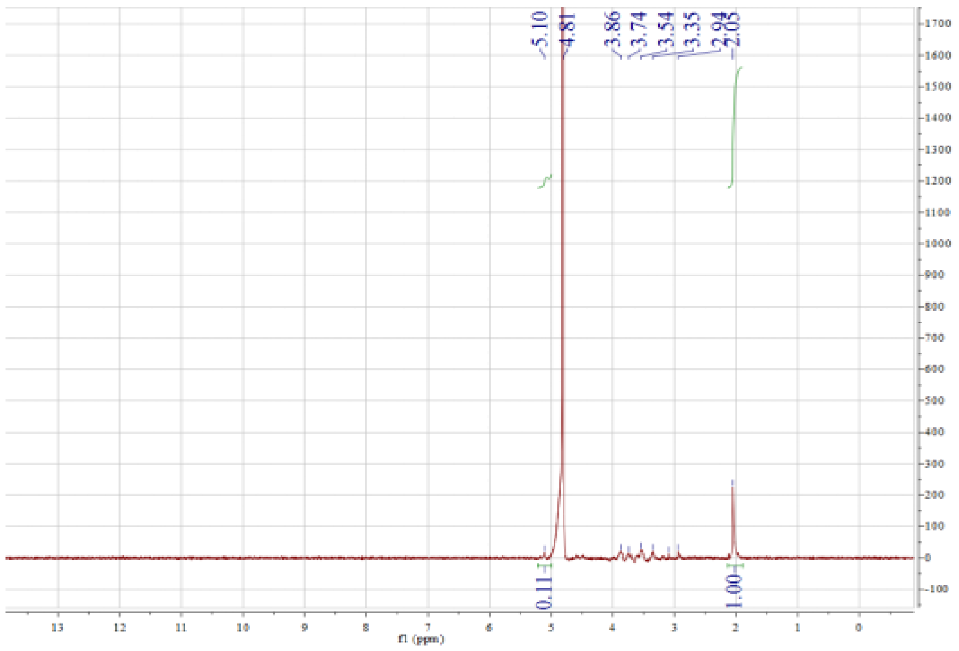

Figure S1 ^1H NMR spectrum (400 MHz, D_2O) of HA-CD (5.1 ppm)

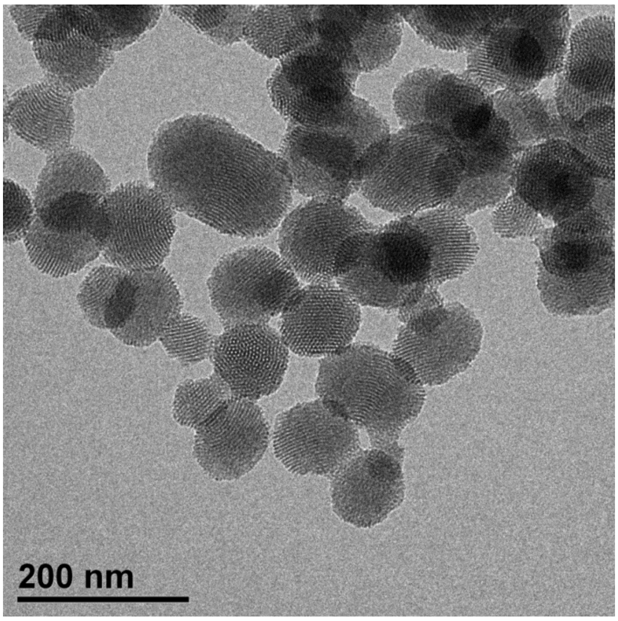

Figure S2 The TEM image of MSN

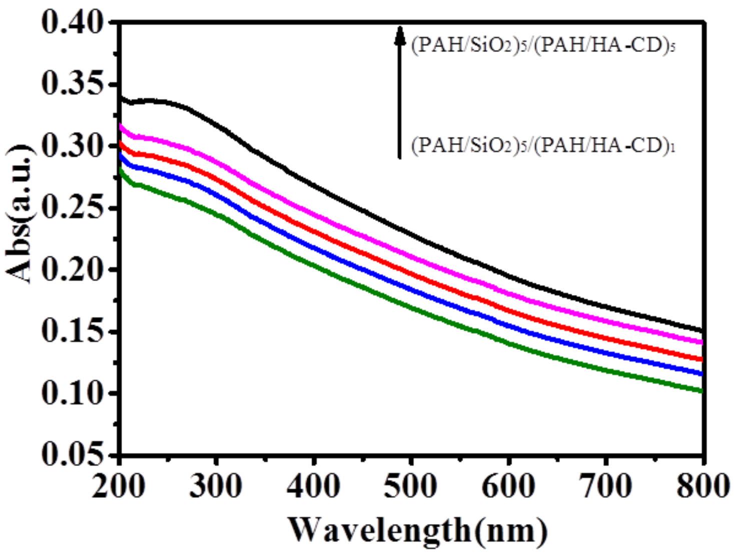

Figure S3 The UV-Vis spectra following the construction of $(PAH/SiO_2)_5/(PAH/HA\text{-}CD)_5$ multilayers

Table S1 Experimental recipe for the preparation of HA/NH_2-β-CD

Product	HA [g]	MES [mL]	EDC [g]	NHS [g]	NH2-β-CD [g]	Grafting rate [%]
HA-CD	0.3	50	0.285	0.342	0.1686	4.71

Table S2 the diffusion coefficient(D) of sodium fluorescein in different concentrations of HA-CD and HA

Gel	Grafting rate	Concentration	diffusion coefficient [μm^2 s^{-1}]
HA	4.71%	3%	34.4338
HA-CD	4.71%	3%	32.4881
HA	4.71%	4%	34.1532
HA-CD	4.71%	4%	28.9492

Table S3 the diffusion coefficient(D) of FITC in different gels of HA-CD and HA

Gel	Grafting rate	Concentration	Diffusion coefficient [μm^2 s^{-1}]
HA	4.71%	3%	30.2861
HA-CD	4.71%	3%	30.2847

Table S4 the release time of fluorescent probe in different kinds of multilayer films

Lamination multilayer films	Fluorescent probe	Release time [min]
$(PAH/SiO_2)_1/(PAH/HA\text{-}CD)_3/(PAH/SiO_2)_1/(PAH/HA\text{-}CD)_3$	FITC-RGD	150
$(PAH/SiO_2)_1/(PAH/HA\text{-}CD)_3/(PAH/SiO_2)_1/(PAH/HA\text{-}CD)_3$	FITC-RGD-Ad	250
$(PAH/SiO_2)_1/(PAH/HA\text{-}CD)_5/(PAH/SiO_2)_1/(PAH/HA\text{-}CD)_5$	FITC-RGD	200
$(PAH/SiO_2)_1/(PAH/HA\text{-}CD)_5/(PAH/SiO_2)_1/(PAH/HA\text{-}CD)_5$	FITC-RGD-Ad	290
$(PAH/SiO_2)_1/(PAH/HA\text{-}CD)_7/(PAH/SiO_2)_1/(PAH/HA\text{-}CD)_7$	FITC-RGD	270
$(PAH/SiO_2)_1/(PAH/HA\text{-}CD)_7/(PAH/SiO_2)_1/(PAH/HA\text{-}CD)_7$	FITC-RGD-Ad	330

Table S5 The release time of fluorescent agent in different kinds of lamination films

Lamination multilayer films	Fluorescent probe	Release time [h]
$(PAH/SiO_2)_1/(PAH/HA\text{-}CD)_{20}/(PAH/SiO_2)_1/(PAH/HA\text{-}CD)_{20}$	FITC-RGD	15
$(PAH/SiO_2)_1/(PAH/HA\text{-}CD)_{20}/(PAH/SiO_2)_1/(PAH/HA\text{-}CD)_{20}$	FITC-RGD-Ad	27
$(PAH/SiO_2)_1/(PAH/HA\text{-}CD)_{30}/(PAH/SiO_2)_1/(PAH/HA\text{-}CD)_{30}$	FITC-RGD	27
$(PAH/SiO_2)_1/(PAH/HA\text{-}CD)_{30}/(PAH/SiO_2)_1/(PAH/HA\text{-}CD)_{30}$	FITC-RGD-Ad	40
$(PAH/SiO_2)_1/(PAH/HA\text{-}CD)_{40}/(PAH/SiO_2)_1/(PAH/HA\text{-}CD)_{40}$	FITC-RGD	40
$(PAH/SiO_2)_1/(PAH/HA\text{-}CD)_{40}/(PAH/SiO_2)_1/(PAH/HA\text{-}CD)_{40}$	FITC-RGD-Ad	55
